# Levulinic acid

**DOI:** 10.1107/S1600536813021090

**Published:** 2013-08-10

**Authors:** Barbara Hachuła, Anna Polasz, Marzena Dzida, Maria Nowak, Joachim Kusz

**Affiliations:** a Institute of Chemistry, University of Silesia, 14 Bankowa Street, 40-006 Katowice, Poland; b Institute of Physics, University of Silesia, 4 Uniwersytecka Street, 40-007 Katowice, Poland

## Abstract

The title compound (systematic name: 4-oxo­penta­noic acid), C_5_H_8_O_3_, is close to planar (r.m.s. deviation = 0.0762 Å). In the crystal, the mol­ecules inter­act *via* O—H⋯O hydrogen bonds in which the hy­droxy O atoms act as donors and the ketone O atoms in adjacent mol­ecules as acceptors, forming *C*(7) chains along [20-1].

## Related literature

For uses of levulinic acid, see: Timokhin *et al.* (1999 ▶). For density functional and Møller–Plesset perturbation theory calculations for levulinic acid, see: Reichert *et al.* (2010 ▶); Kim *et al.* (2011 ▶). For typical bond lengths and angles, see: Allen *et al.* (1987 ▶); Borthwick (1980 ▶). For hydrogen-bond motifs, see: Bernstein *et al.* (1995 ▶); Etter *et al.* (1990 ▶). For background to the study, see: Flakus & Hachuła (2008 ▶); Flakus & Stachowska (2006 ▶).
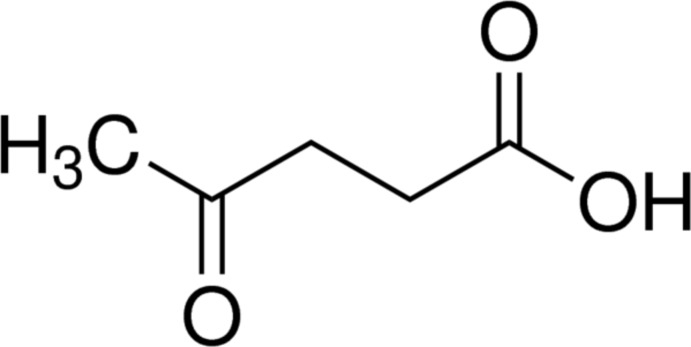


## Experimental

### 

#### Crystal data

C_5_H_8_O_3_
*M* *_r_* = 116.11
Monoclinic, 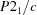

*a* = 4.8761 (2) Å
*b* = 12.1025 (4) Å
*c* = 9.8220 (3) Å
β = 99.112 (3)°
*V* = 572.31 (3) Å^3^
*Z* = 4
Mo *K*α radiation
μ = 0.11 mm^−1^
*T* = 100 K
0.44 × 0.21 × 0.16 mm

#### Data collection

Oxford Diffraction Xcalibur diffractometer with a Sapphire3 detector
Absorption correction: multi-scan (*CrysAlis RED*; Oxford Diffraction, 2006 ▶) *T*_min_ = 0.585, *T*_max_ = 1.000
7178 measured reflections
1013 independent reflections
902 reflections with *I* > 2σ(*I*)
*R*_int_ = 0.034

#### Refinement

*R*[*F* ^2^ > 2σ(*F* ^2^)] = 0.040
*wR*(*F* ^2^) = 0.114
*S* = 1.06
1013 reflections
77 parameters
H atoms treated by a mixture of independent and constrained refinement
Δρ_max_ = 0.23 e Å^−3^
Δρ_min_ = −0.24 e Å^−3^

### 

Data collection: *CrysAlis CCD* (Oxford Diffraction, 2006 ▶); cell refinement: *CrysAlis RED* (Oxford Diffraction, 2006 ▶); data reduction: *CrysAlis RED*; program(s) used to solve structure: *SHELXS97* (Sheldrick, 2008 ▶); program(s) used to refine structure: *SHELXL97* (Sheldrick, 2008 ▶); molecular graphics: *Mercury* (Macrae *et al.*, 2006 ▶); software used to prepare material for publication: *publCIF* (Westrip, 2010 ▶).

## Supplementary Material

Crystal structure: contains datablock(s) I. DOI: 10.1107/S1600536813021090/ff2114sup1.cif

Structure factors: contains datablock(s) I. DOI: 10.1107/S1600536813021090/ff2114Isup2.hkl

Supplementary material file. DOI: 10.1107/S1600536813021090/ff2114Isup3.cml

Additional supplementary materials:  crystallographic information; 3D view; checkCIF report

## Figures and Tables

**Table 1:** Hydrogen-bond geometry (Å, °)

*D*—H⋯*A*	*D*—H	H⋯*A*	*D*⋯*A*	*D*—H⋯*A*
O1—H1⋯O3^i^	0.83 (2)	1.87 (2)	2.6977 (13)	176 (2)

Symmetry code: (i) 
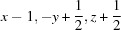
.

**Fig. 1.**
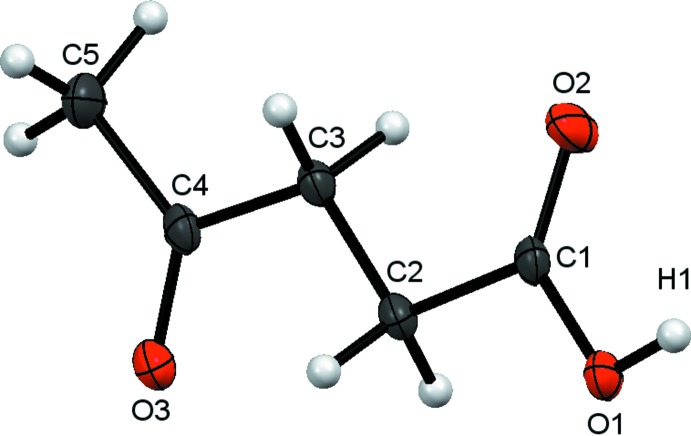
The asymmetric unit of (I), with the atom-numbering scheme, showing 50% probability displacement ellipsoids. H atoms are shown as small spheres of arbitrary radius.

**Fig. 2.**
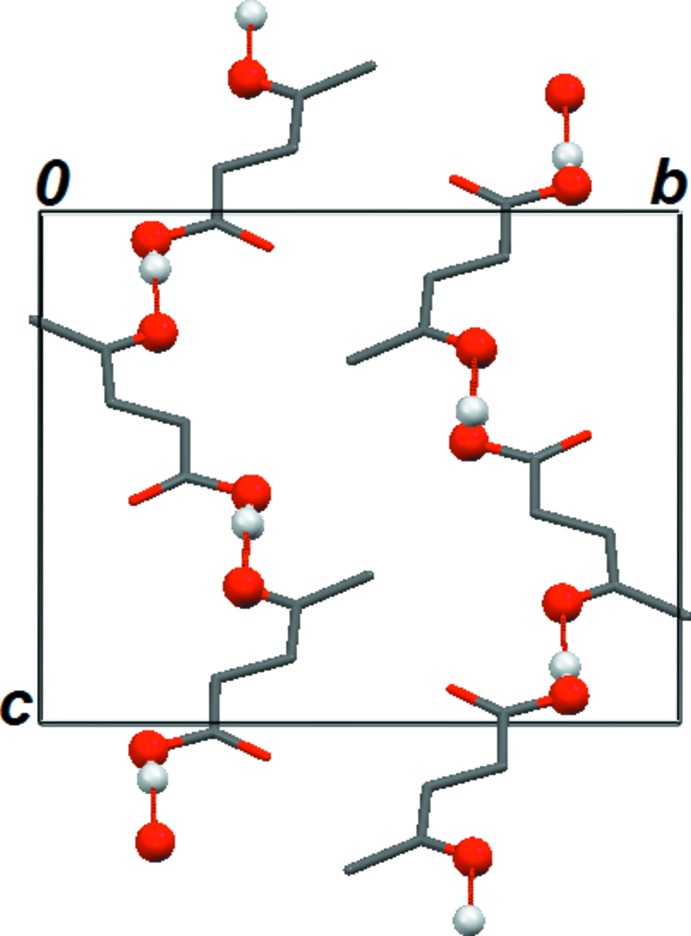
Part of the crystal structure of (I), viewed along the a axis, showing the C(7) chains. The red lines indicate the hydrogen-bonding interactions. For the sake of clarity, all H atoms bonded to C atoms were omitted.

## supplementary crystallographic information

### 1. Comment

Levulinic acid [systematic name: 4-oxopentanoic acid], (I), is a biogenic product of hexose acid hydrolysis at elevated temperatures that can be obtained from renewable resources. This functionalized carbon structure is widely used as chemical intermediate in the manufacture of fuel extenders, biodegradable polymers, herbicides, antibiotics, flavours and useful 5-carbon compounds (Timokhin *et al.*, 1999; Reichert *et al.*, 2010). Levulinic acid was investigated in a continuation of our studies of the IR spectra of hydrogen bonding in carboxylic acid derivatives (Flakus & Stachowska, 2006; Flakus & Hachuła, 2008). In order to study interactions occurring *via* hydrogen bonds and molecular packing in this compound, we have now determined the structure of (I) using diffraction data collected at 100 K.

The molecule of (I) is nearly planar (r.m.s. deviation of fitted all non-hydrogen atoms is equal to 0.0762 Å). The C—O (1.3373 (17) Å) and C=O (1.2044 (17) Å) bond distances differ slightly from the mean values given by Allen *et al.* (1987) for a variety of carboxylic acid groups (C—O 1.308 Å and C=O 1.214 Å). The bond-angle values at the central C atom in the carboxylic acid group of (I) (O2—C1—C2 124.51 (13) °; O1—C1—C2 112.48 (12)°) agree well with the mean values specified by Borthwick (1980) for a typical carboxylic acid group (O2—C1—C2 123 (2)°; O1—C1—C2 112 (2)°).

The monoclinic structure of (I) is composed of molecular sheets stacked along [101] direction. Atom O1 of the carboxylic group acts as a hydrogen-bond donor *via* H1 to carbonyl atom O3 belonging to the acetyl group of adjacent molecule. This interaction generates hydrogen-bonded chain with a graph-set motif of *C*(7) (Etter *et al.*, 1990; Bernstein *et al.*, 1995).

### 2. Experimental

Levulinic acid was purchased from Aldrich-Sigma. Crystals of title compound, suitable for X-ray diffraction, were selected directly from purchased sample.

### 3. Refinement

The H atoms were introduced in geometrically idealized positions and allowed for with an appropriate riding model with C—H distances of 0.99 Å (CH_2_) and *U*_iso_(H) values set at 1.2*U*_eq_(C) or 0.98 Å (CH_3_) and with *U*_iso_(H) values set at 1.5*U*_eq_(C). The H atom which takes part in hydrogen bonding was located in a difference Fourier map and was refined with *U*_iso_(H) value set at 1.5*U*_eq_(O).

### Crystal data

**Table:**

C_5_H_8_O_3_	*F*(000) = 248
*M**_r_* = 116.11	*D*_x_ = 1.348 Mg m^−^^3^
Monoclinic, *P*2_1_/*c*	Melting point = 303–306 K
Hall symbol: -P 2ybc	Mo *K*α radiation, λ = 0.71073 Å
*a* = 4.8761 (2) Å	Cell parameters from 6623 reflections
*b* = 12.1025 (4) Å	θ = 3.4–34.5°
*c* = 9.8220 (3) Å	µ = 0.11 mm^−^^1^
β = 99.112 (3)°	*T* = 100 K
*V* = 572.31 (3) Å^3^	Polyhedron, colourless
*Z* = 4	0.44 × 0.21 × 0.16 mm

### Data collection

**Table:**

Oxford Diffraction Xcalibur diffractometer with a Sapphire3 detector	1013 independent reflections
Radiation source: fine-focus sealed tube	902 reflections with *I* > 2σ(*I*)
Graphite monochromator	*R*_int_ = 0.034
Detector resolution: 16.0328 pixels mm^-1^	θ_max_ = 25.1°, θ_min_ = 3.4°
ω scan	*h* = −5→4
Absorption correction: multi-scan (*CrysAlis RED*; Oxford Diffraction, 2006)	*k* = −14→14
*T*_min_ = 0.585, *T*_max_ = 1.000	*l* = −11→11
7178 measured reflections	

### Refinement

**Table:**

Refinement on *F*^2^	Primary atom site location: structure-invariant direct methods
Least-squares matrix: full	Secondary atom site location: difference Fourier map
*R*[*F*^2^ > 2σ(*F*^2^)] = 0.040	Hydrogen site location: inferred from neighbouring sites
*wR*(*F*^2^) = 0.114	H atoms treated by a mixture of independent and constrained refinement
*S* = 1.06	*w* = 1/[σ^2^(*F*_o_^2^) + (0.0797*P*)^2^ + 0.128*P*] where *P* = (*F*_o_^2^ + 2*F*_c_^2^)/3
1013 reflections	(Δ/σ)_max_ < 0.001
77 parameters	Δρ_max_ = 0.23 e Å^−^^3^
0 restraints	Δρ_min_ = −0.24 e Å^−^^3^

### Special details

**Table:**

Experimental. CrysAlis RED (Oxford Diffraction, 2006). Empirical absorption correction using spherical harmonics, implemented in SCALE3 ABSPACK scaling algorithm.
Geometry. All e.s.d.'s (except the e.s.d. in the dihedral angle between two l.s. planes) are estimated using the full covariance matrix. The cell e.s.d.'s are taken into account individually in the estimation of e.s.d.'s in distances, angles and torsion angles; correlations between e.s.d.'s in cell parameters are only used when they are defined by crystal symmetry. An approximate (isotropic) treatment of cell e.s.d.'s is used for estimating e.s.d.'s involving l.s. planes.
Refinement. Refinement of *F*^2^ against ALL reflections. The weighted *R*-factor *wR* and goodness of fit *S* are based on *F*^2^, conventional *R*-factors *R* are based on *F*, with *F* set to zero for negative *F*^2^. The threshold expression of *F*^2^ > σ(*F*^2^) is used only for calculating *R*-factors(gt) *etc*. and is not relevant to the choice of reflections for refinement. *R*-factors based on *F*^2^ are statistically about twice as large as those based on *F*, and *R*- factors based on ALL data will be even larger.

### Fractional atomic coordinates and isotropic or equivalent isotropic displacement parameters (Å^2^)

**Table:**

	*x*	*y*	*z*	*U*_iso_*/*U*_eq_	
O1	0.4194 (2)	0.16988 (8)	1.05179 (10)	0.0186 (3)	
O2	0.3930 (2)	0.35384 (9)	1.06449 (12)	0.0286 (4)	
O3	1.0589 (2)	0.31839 (8)	0.73324 (10)	0.0197 (3)	
C1	0.4836 (3)	0.27238 (11)	1.01688 (14)	0.0159 (4)	
C2	0.6782 (3)	0.27359 (12)	0.91177 (14)	0.0164 (4)	
H2A	0.5932	0.2323	0.8288	0.020*	
H2B	0.8537	0.2361	0.9505	0.020*	
C3	0.7411 (3)	0.39098 (12)	0.87113 (14)	0.0170 (4)	
H3A	0.5641	0.4276	0.8328	0.020*	
H3B	0.8221	0.4319	0.9552	0.020*	
C4	0.9364 (3)	0.39950 (11)	0.76733 (13)	0.0163 (4)	
C5	0.9722 (3)	0.51213 (13)	0.70857 (16)	0.0242 (4)	
H5A	1.1394	0.5130	0.6648	0.036*	
H5B	0.9909	0.5671	0.7827	0.036*	
H5C	0.8097	0.5299	0.6398	0.036*	
H1	0.311 (4)	0.1766 (16)	1.108 (2)	0.036*	

### Atomic displacement parameters (Å^2^)

**Table:**

	*U*^11^	*U*^22^	*U*^33^	*U*^12^	*U*^13^	*U*^23^
O1	0.0253 (6)	0.0186 (6)	0.0137 (5)	−0.0011 (4)	0.0093 (4)	0.0006 (4)
O2	0.0435 (7)	0.0204 (6)	0.0275 (7)	0.0010 (5)	0.0231 (5)	−0.0016 (5)
O3	0.0240 (6)	0.0218 (6)	0.0148 (5)	0.0025 (4)	0.0072 (4)	0.0001 (4)
C1	0.0200 (7)	0.0187 (7)	0.0088 (7)	−0.0002 (6)	0.0020 (5)	0.0003 (5)
C2	0.0202 (7)	0.0187 (8)	0.0112 (7)	0.0017 (5)	0.0050 (5)	−0.0009 (5)
C3	0.0218 (7)	0.0183 (8)	0.0118 (7)	−0.0002 (5)	0.0060 (6)	−0.0015 (5)
C4	0.0183 (7)	0.0207 (8)	0.0092 (7)	0.0000 (5)	0.0001 (5)	−0.0016 (6)
C5	0.0334 (8)	0.0224 (8)	0.0195 (8)	0.0008 (6)	0.0125 (6)	0.0036 (6)

### Geometric parameters (Å, º)

**Table:**

O1—C1	1.3373 (17)	C3—C4	1.5050 (19)
O1—H1	0.83 (2)	C3—H3A	0.9900
O2—C1	1.2044 (17)	C3—H3B	0.9900
O3—C4	1.2231 (17)	C4—C5	1.501 (2)
C1—C2	1.5092 (19)	C5—H5A	0.9800
C2—C3	1.520 (2)	C5—H5B	0.9800
C2—H2A	0.9900	C5—H5C	0.9800
C2—H2B	0.9900		
			
C1—O1—H1	106.3 (14)	C4—C3—H3B	108.6
O2—C1—O1	123.01 (13)	C2—C3—H3B	108.6
O2—C1—C2	124.51 (13)	H3A—C3—H3B	107.6
O1—C1—C2	112.48 (12)	O3—C4—C5	122.14 (13)
C1—C2—C3	111.32 (12)	O3—C4—C3	121.30 (12)
C1—C2—H2A	109.4	C5—C4—C3	116.57 (12)
C3—C2—H2A	109.4	C4—C5—H5A	109.5
C1—C2—H2B	109.4	C4—C5—H5B	109.5
C3—C2—H2B	109.4	H5A—C5—H5B	109.5
H2A—C2—H2B	108.0	C4—C5—H5C	109.5
C4—C3—C2	114.68 (12)	H5A—C5—H5C	109.5
C4—C3—H3A	108.6	H5B—C5—H5C	109.5
C2—C3—H3A	108.6		
			
O2—C1—C2—C3	−1.2 (2)	C2—C3—C4—O3	−8.66 (18)
O1—C1—C2—C3	178.42 (10)	C2—C3—C4—C5	171.36 (11)
C1—C2—C3—C4	179.46 (11)		

### Hydrogen-bond geometry (Å, º)

**Table:**

*D*—H···*A*	*D*—H	H···*A*	*D*···*A*	*D*—H···*A*
O1—H1···O3^i^	0.83 (2)	1.87 (2)	2.6977 (13)	176 (2)

Symmetry code: (i) *x*−1, −*y*+1/2, *z*+1/2.
